# Development of DNA aptamers targeting B7H3 by hybrid-SELEX: an alternative to antibodies for immuno-assays

**DOI:** 10.1038/s41598-024-64559-7

**Published:** 2024-06-12

**Authors:** Bhavani Shankar Maradani, Sowmya Parameswaran, Krishnakumar Subramanian

**Affiliations:** 1 L&T Ocular Pathology Department, Vision Research Foundation, Sankara Nethralaya, No. 41, College Road, Nungambakkam, Chennai, Tamil Nadu 600006 India; 2 Radheshyam Kanoi Stem Cell Laboratory, Vision Research Foundation, Chennai, India; 3 School of Chemical and Biotechnology, SASTRA Deemed University, Thanjavur, India

**Keywords:** Biological techniques, Biotechnology, Cancer, Biomarkers

## Abstract

Antibodies have been extensively used in numerous applications within proteomics-based technologies, requiring high sensitivity, specificity, a broad dynamic range for detection, and precise, reproducible quantification. Seeking alternatives to antibodies due to several inherent limitations of antibodies is an area of active research of tremendous importance. Recently, aptamers have been receiving increasing attention, because they not only have all of the advantages of antibodies, but also have unique advantages, such as thermal stability, low cost, and unlimited applications. Aptamers are gaining importance in immunological studies and can potentially replace antibodies in immunoassays. B7H3, an immunoregulatory protein belonging to the B7 family, is an attractive and promising target due to its overexpression in several tumor tissues while exhibiting limited expression in normal tissues. This study employed hybrid-SELEX with next-generation sequencing to select ssDNA aptamers specifically binding to the B7H3 protein. These aptamers demonstrated versatility across various assays, including flow cytometry, dot-blot, and immunohistochemistry. Effective performance in sandwich dot-blot assays and western blot analysis suggests their potential for diagnostic applications and demonstrates their adaptability and cost-effectiveness in diverse protein detection techniques.

## Introduction

Antibodies are one of the most important indispensable tools in protein research, due to their high specificity, affinity, and selectivity. Antibodies have proven to be very effective affinity ligands of choice for identifying proteins in research, diagnostics, biosensors, imaging, and therapeutics^1^. Routine procedures such as flow cytometry, immunohistochemistry (IHC), western blot, enzyme-linked immunosorbent assay (ELISA), and many others all rely on antibodies. Antibodies are broadly categorized into polyclonal, monoclonal, and recombinant antibodies. The heterogeneous nature of polyclonal antibodies makes them more prone to batch-to-batch variability and cross-reactivity with other molecules resulting in a higher background. Even though monoclonal and recombinant antibodies are highly selective high-affinity binding ligands, homogenous between the batches, they have limitations such as selection difficulties, selectivity problems, high costs of production, and stability^2,3^. Better molecular probes that can circumvent these inconsistencies, with sensitivity and specificity (with limited cross-reactivity) and stability, affording a long half-life are needed.

Nucleic acid aptamers colloquially known as ‘chemical antibodies’ are emerging as attractive alternatives to antibodies and small molecules in diagnostic, therapeutic, imaging, and targeting applications^4–8^. These aptamers are nucleic acids (DNA or RNA) with tertiary conformations that have sufficient surface area to recognize and bind their targets via an induced fit mechanism, much like antibodies^9^, and exhibit high selectivity and binding affinity with dissociation constants typically in the nanomolar (nM) to picomolar (pM) range. Aptamers offer significant advantages over antibodies being sequence defined, simple with longer shelf life, thermally stable, chemically modified, highly consistent with no batch-to-batch variation, cost-effective, and the time required for aptamers generation is comparatively short^6,10^. Aptamers are selected through an in-vitro method, a process that involves iterative cycles of incubating a randomized nucleic acid library with the target of interest, known as Systematic Evolution of Ligands by Exponential Enrichment (SELEX). The SELEX process's iterative nature, combined with its inherent strictness, leads to the creation of aptamers that demonstrate greater specificity compared to equivalent antibodies.

Several studies have compared aptamers to antibodies to detect proteins in immuno-assays. Aptamers have replaced antibodies in histochemical staining of both fresh-frozen and paraffin-embedded tissue sections^11–14^, flow-cytometry^14–17^, dot-blot^18,19^, western blot^20,21^, ELISA^22,23^ and many other antibody-dependent assays. All the above studies are about the application of aptamers in comparison antibodies to a single assay. To date, no detailed comprehensive studies have validated the aptamers for multiple protein detection assays and reported an aptamer that can be applied for multiple assays. In the present study, we have developed ssDNA aptamers against B7H3 for studying the application of aptamers for various assays as an alternative to antibodies.

We have chosen B7H3 as the potential target for the comparison of antibody Vs. aptamer due to the following reasons:

1. B7-H3 has been a clinically significant therapeutic target for anti-cancer therapy due to its overexpression in various tumours^24,25^.
2. Expression of B7H3 at various localisations (membranous and secretory) which aids better validation^25^.
3. With our previous studies on the variable expression patterns of B7H3 in Retinoblastoma tumors^26^.
4. Availability of commercial antibodies for comparison with the developed aptamers.

In this study, we used hybrid-SELEX with next-generation sequencing to select ssDNA aptamer sequences specifically bound to the B7H3 protein. We have evaluated the application of the developed aptamers for various protein detection assays in comparison with the antibody. The results indicate that the obtained aptamers can specifically recognize B7H3 and hold great potential to be used as a molecular recognition probe in flow-cytometry, IHC, dot-blot, sandwich dot-blot, and western blotting techniques.

## Results

### Pre-screening of RB Cell-SELEX enriched aptamer pools by dot-blot

**Figure 1:**
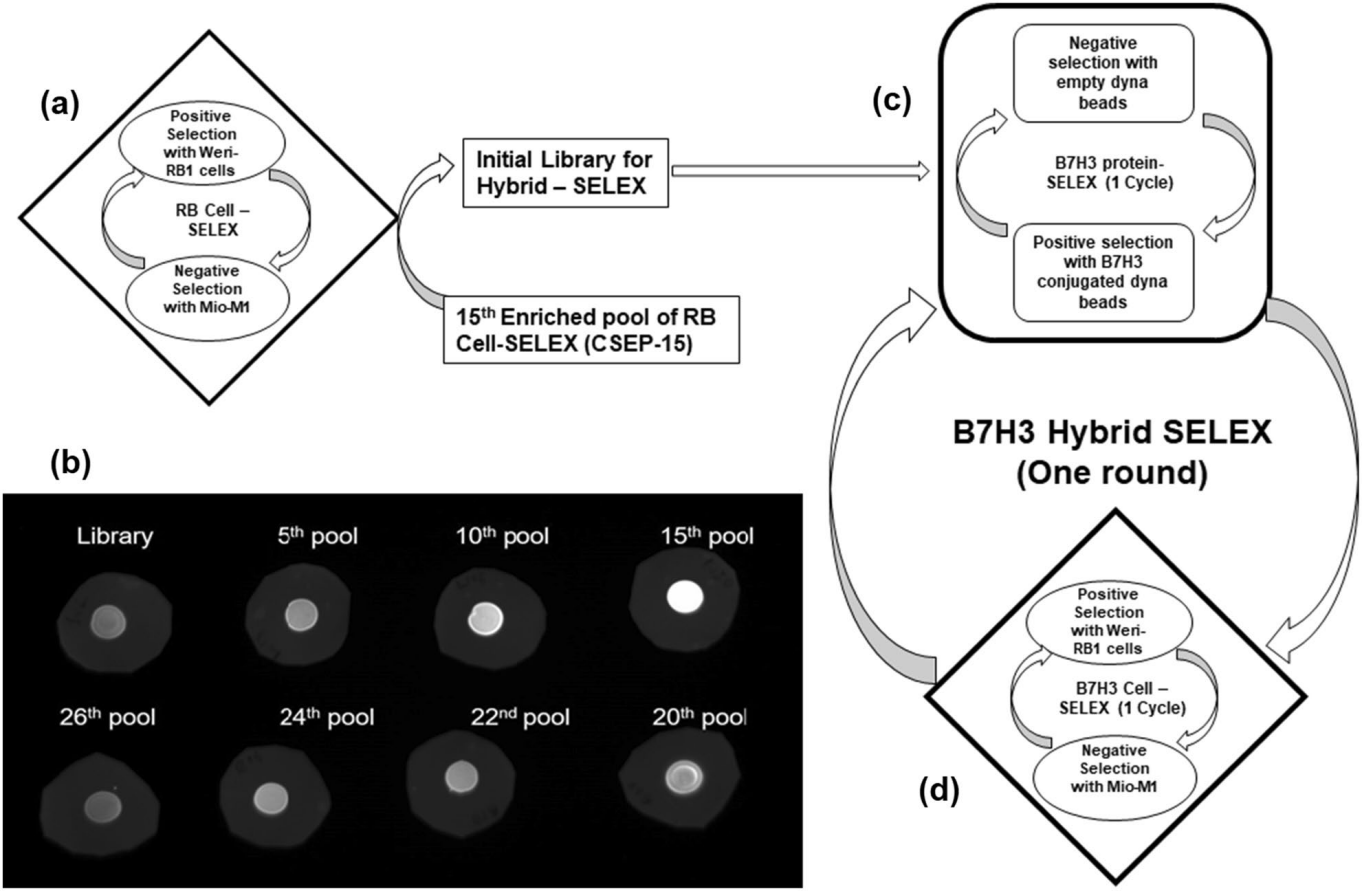
Schematic representation of our hybrid-SELEX method for selection of B7H3-specific ssDNA aptamer. (**a**) Retinoblastoma cell-SELEX to develop aptamers against retinoblastoma using Weri-RB1 cells and target cell and Mio-M1 as control cells. (**b**) We have screened the RB cell-SELEX enriched pools on recombinant B7H3 protein by dot-blot and chose the cell-SELEX enriched pool-15 (CSEP-15) as the starting library for the B7H3 hybrid SELEX. In our experiment, the hybrid-SELEX process is divided into (**c**) the B7H3 protein-based SELEX selection and (**d**) cell-based SELEX enrichment. The CSEP-15 is incubated with B7H3 protein immobilized on magnetic beads for positive selection and empty magnetic beads for counter selection for each cycle in protein-SELEX. The pool enriched from protein SELEX is incubated with Weri-RB1 for positive selection and Mio-M1 for counter selection in cell-SELEX. After 9 rounds of selection, the enriched aptamer pools were sequenced by NGS. SELEX, Systematic Evolution of Ligands by EXponential enrichment.

We have screened the previously enriched RB cell-SELEX pools (Fig. 1a) for the binding affinity with the recombinant B7H3 protein by dot blot. The pools showed significant binding to the B7H3 protein with the maximum fluorescence intensity for the 15th enriched pool which diminished further with the higher enriched pools (Fig. 1b). Based on these pre-screening results, we chose the 15th RB cell-SELEX enriched pool (CSEP-15) as the starting library for the process of B7H3 hybrid SELEX. Further, we have selected the Weri-RB1 and Mio-M1 for the cell-SELEX part of B7H3 hybrid-SELEX.

### Selection of DNA aptamers against B7H3 through Hybrid SELEX

In our screening process, to obtain B7H3 aptamers we combined two selection strategies, protein-SELEX and cell-SELEX (Fig. 1c,d). One cycle of protein-SELEX followed by one cycle of cell-SELEX is considered as one round of selection. In the first strategy, protein-SELEX, where we employed high-affinity interactions between histidine tags at the C-terminus of recombinant B7H3 protein with cobalt-based dyna-beads to enrich B7H3 binding aptamers. There is a possibility of obtaining non-specific dyna-beads binding aptamers, therefore we used the dyna-beads as a negative control while the B7H3-beads were used as the positive target in SELEX to remove the non-specific surface binding sequences. During each cycle of protein-SELEX, PCR-amplified dsDNA was identified and passed through the affinity purification on streptavidin-sepharose beads followed by alkaline separation to get FITC-labelled ssDNA, which was used as an input for the sequential cycle of cell-SELEX. Cell-SELEX was performed using Weri-RB1 cells for positive selection and Mio-M1 for negative selection as described in Maradani et al.^14^. The enriched ssDNA of the cell-SELEX cycle was used as input for the next cycle of protein-SELEX of successive round of the selection.

**Figure 2:**
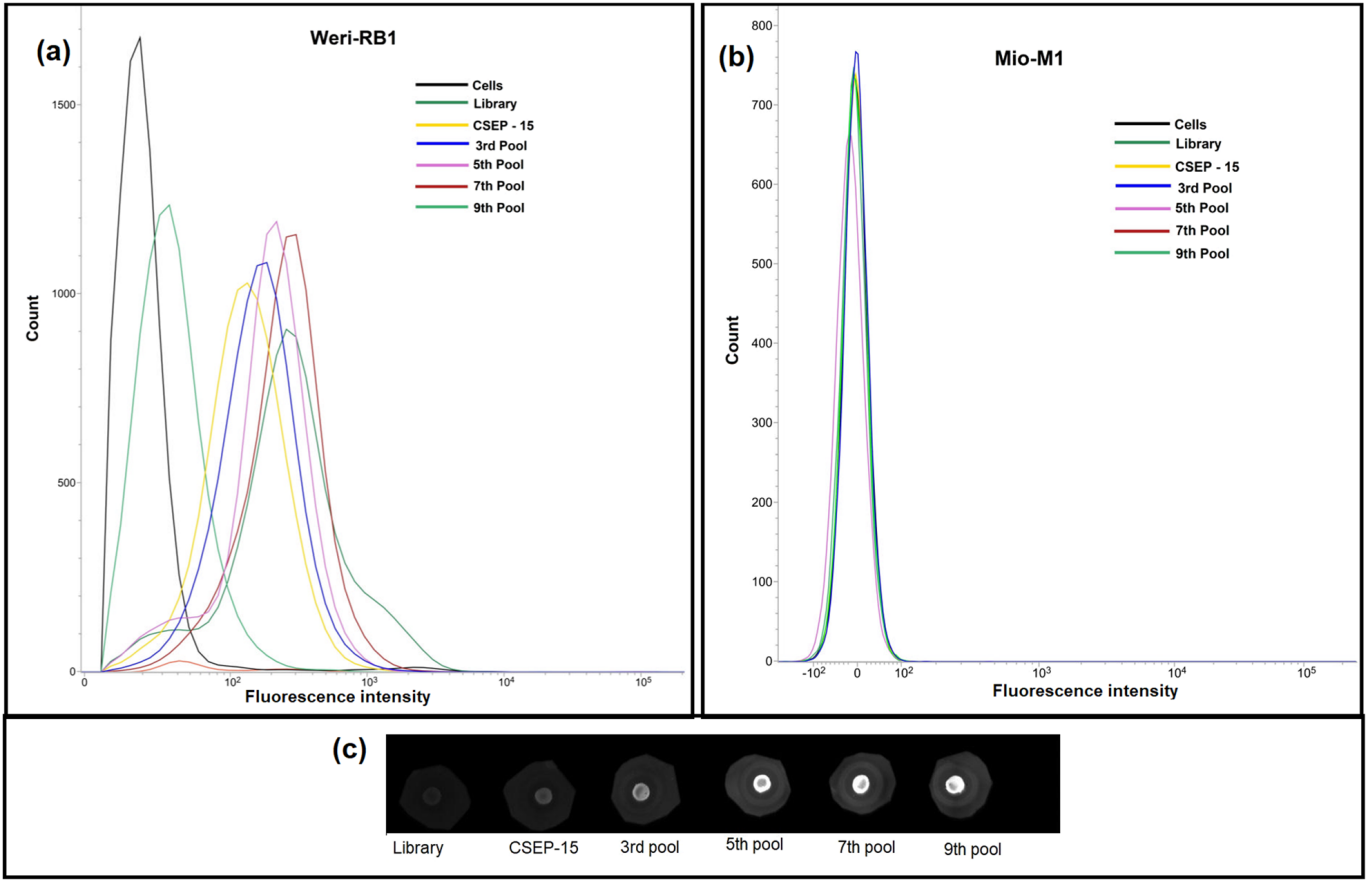
Monitoring the enrichment of the DNA libraries during hybrid-SELEX by flow cytometry and dot-blot. (**a**) Fluorescence intensities of target cells (Weri-RB1) incubated with FITC-labelled ssDNA pools from the initial library to the ninth-selection round. (**b**) Fluorescence intensities of negative control cells (Mio-M1) incubated with FITC-labelled ssDNA pools from the initial library to the ninth-selection round. (**c**) Represented is the dot-blot assay showing the fluorescence intensities of ssDNA pools from the initial library to the ninth-selection round, bound to the recombinant B7H3 protein.

To further evaluate our protocol, the enrichment of aptamers was monitored on Weri-RB1 and Mio-M1 cells by flow cytometry. The initial ssDNA library, CSEP-15, 3, 5, 7, and 9th round pools were tested for selection enrichment. We found that enrichment did not substantially increase in rounds 1–2 (data not shown) and after the 7th round of selection, but increased in between rounds (rounds 3–9). As shown in Fig. 2a,b, with the progression of selection cycles, there was a steady increase in the fluorescence intensity on the Weri-RB1 cells till the 7th cycle, whereas there was no obvious change in fluorescence intensity on the Mio-M1 cells. The enrichment was also monitored by checking the binding of enriched pools on the recombinant B7H3 protein by dot-bot (Fig. 2c). The increase in the fluorescent intensity by binding of the enriched pools to the recombinant protein, with the progression of the SELEX rounds further confirmed the enrichment. The enriched pools were sequenced by Illumina sequencing at Base-pair Biotechnologies, Texas (US).

Based on the abundance in the sequenced pools (Fig. S1) and homology within their variable core region, the top 10 most frequent oligonucleotide sequences were shortlisted. Among the 10 aptamer sequences, five candidates were chosen based on the percentage of abundance in the sequenced pools (Fig. S2b) for further validation. Then, the selected aptamers were synthesized with FITC and biotin labels for characterization (Table 1).

**Table 1:** Sequences of the top five B7H3 aptamers selected for characterization.

Aptamer name	Sequence (5ʹ–3ʹ)
VRF-HS_B7H3-01	TAGGGAAGAGAAGGACATATGATACAGCACGGTATGACGAGTCCATTGTGTGTGCAGCTCGCTAATTGACTAGTACATGACCACTT
VRF-HS_B7H3-02	TAGGGAAGAGAAGGACATATGATCGCCCCTCTTTTCGATTTCAAGCTCCTAACCCACACCTCTTTGACTAGTACATGACCACTT
VRF-HS_B7H3-03	TAGGGAAGAGAAGGACATATGATATAAGTGACACATCAATCTCATGCGGATGAACCAGTGCGTTTGACTAGTACATGACCACTT
VRF-HS_B7H3-04	TAGGGAAGAGAAGGACATATGATTGCCCCTGACACTTACACGCCACTATCAAGGCACACGCCATTGACTAGTACATGACCACTT
VRF-HS_B7H3-05	TAGGGAAGAGAAGGACATATGATTGAGAAGTCACGCCACTTACCTGCGCTCAAAACCTCAGAATTGACTAGTACATGACCACTT

#### Binding affinity of aptamers

**Figure 3:**
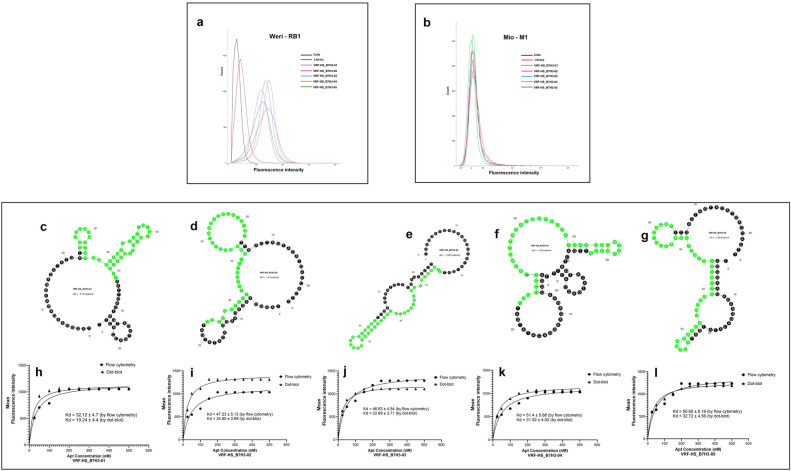
Binding ability, secondary structures and dissociation constants of selected B7H3 aptamers. (**a**) Binding affinity of FITC-labelled aptamers VRF-HS_B7H3-01, VRF-HS_B7H3-02, VRF-HS_B7H3-03, VRF-HS_B7H3-04 and VRF-HS_B7H3-05 to Weri-RB1 cells assessed by flow cytometry. (**b**) Binding ability of FITC-labelled aptamers VRF-HS_B7H3-01, VRF-HS_B7H3-02, VRF-HS_B7H3-03, VRF-HS_B7H3-04 and VRF-HS_B7H3-05 to Mio-M1 cells assessed by flow cytometry. (**c–g**) Predicted secondary structures for five aptamer candidates, VRF-HS_B7H3-01, VRF-HS_B7H3-02, VRF-HS_B7H3-03, VRF-HS_B7H3-04 and VRF-HS_B7H3-05 selected for further. The presented predicted secondary structures were the ones with lowest ΔG. Constant sequence regions are highlighted in black, and green represents the random regions. (**h–l**) Binding curve of aptamers VRF-HS_B7H3-01, VRF-HS_B7H3-02, VRF-HS_B7H3-03, VRF-HS_B7H3-04 and VRF-HS_B7H3-05 with Weri-RB1 and Mio-M1 cells assessed by flow cytometry and recombinant B7H3 protein by dot-blot. Equilibrium dissociation constants (Kd) (nM) were calculated using GraphPad Prism 7, under the non-linear fit model, one-site non-competitive binding to fluorescent population ratio at used aptamer concentrations.

The binding assay of the selected aptamers on the target and control cells was performed by flow cytometry. The fluorescence shift of FITC-aptamers binding to Weri-RB1 cells demonstrated that all five aptamers bound to target cells with high affinity and had no specific binding to Mio-M1 cells. The five aptamers bound to approximately 96%, 92%, 97%, 90%, and 94% of the overall Weri-RB1 population respectively, which was comparable to the percentage of the Weri-RB1 cells bound to the antibody (Fig. S3), with almost no positive signal detected for Mio-M1 cells (Fig. 3a,b). To calculate the Kd values, binding-affinity assays of the selected aptamers were performed by flow cytometry on the Weri-RB1 cell line and by dot-blot on the recombinant B7H3 protein. Figure 3c–g shows the secondary structures of the 5 aptamers and Fig. 3h–l shows the respective binding saturation curves with the target Weri-RB1 cell line and recombinant B7H3 protein, leading to Kd values of 32.12 ± 4.7 and 19.24 ± 4.4 nM for VRF-HS_B7H3-01, 47.23 ± 5.15 and 24.60 ± 3.69 nM for VRF-HS_B7H3-02, 46.63 ± 4.54 and 22.69 ± 3.71 nM for VRF-HS_B7H3-03, 51.40 ± 5.68 and 31.52 ± 4.02 nM for VRF-HS_B7H3-04, and 50.60 ± 6.19 and 32.72 ± 4.56 nM for VRF-HS_B7H3-05, by flow cytometry and dot-blot respectively.

#### Dot Blot

**Figure 4:**
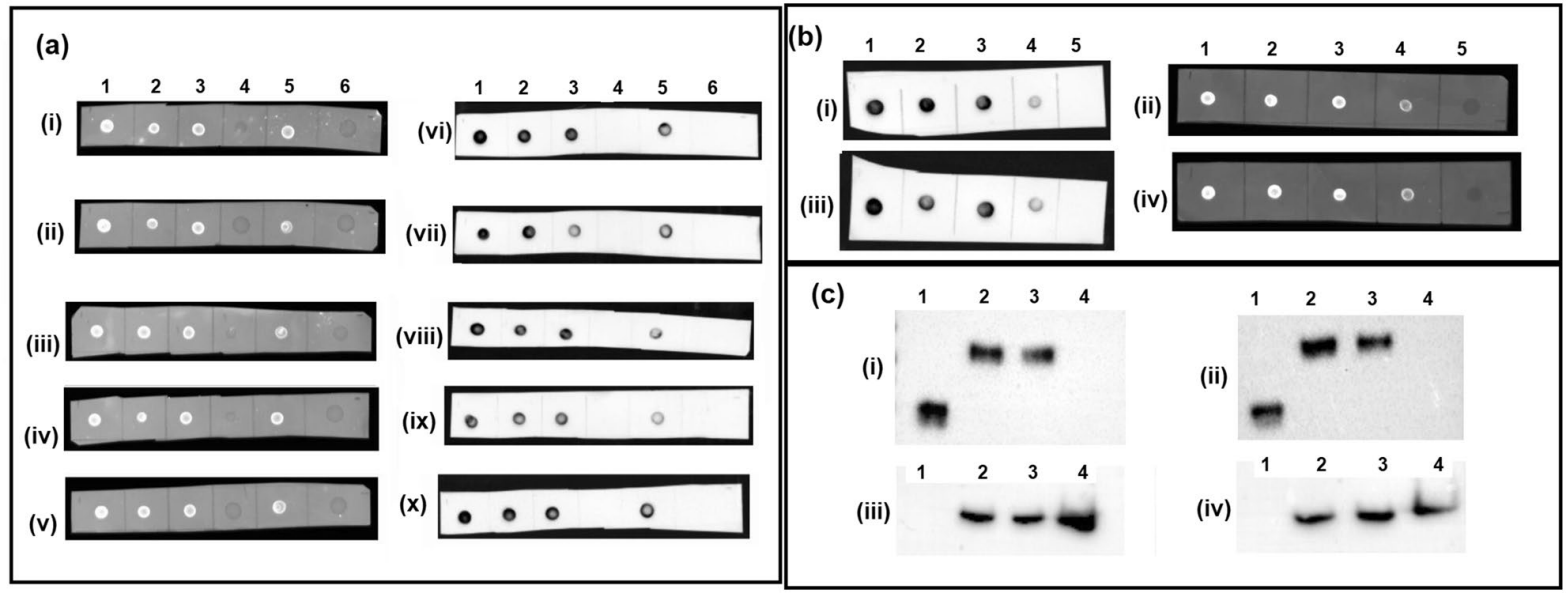
Affinity of B7H3 aptamers by Dot-blot and western blot analysis. (**a**) Dot-blot assay with (i–v) FITC labelled and (vi–x) biotin labelled aptamers to demonstrate the capability of the B7H3 aptamers to recognize their target immobilized on PVDF membranes; (i) & (vi)—VRF-HS_B7H3-01, (ii) & (vii)—VRF-HS_B7H3-02, (iii) & (viii)—VRF-HS_B7H3-03, (iv) & (viii)—VRF-HS_B7H3-04 and (v) & (x)—VRF-HS_B7H3-05; 1—B7H3 Recombinant protein, 2—RB tumor protein lysate, 3—Weri-RB1 protein lysate, 4—BSA, 5—Weri-RB1 secretome and 6—Secondary control. (**b**) Sandwich dot blot assay with (i–iii) biotin labelled and (ii–iv) FITC labelled aptamers to demonstrate the capability of aptamers to recognize different epitopes of their target immobilized on nitrocellulose membranes. (i) & (ii)—unlabelled VRF-HS_B7H3-01 is used as capture and FITC or biotin labelled VRF-HS_B7H3-03 is used for detection, (iii) & (iv) unlabelled VRF-HS_B7H3-03 is used as capture and FITC or biotin labelled VRF-HS_B7H3-01 is used for detection; 1—B7H3 Recombinant protein, 2—RB tumor protein lysate, 3—Weri-RB1 protein lysate, 4—Weri-RB1 secretome and 5—BSA. (**c**) Comparison of the specificity of B7H3 antibody to VRF-HS_B7H3-03 aptamer in a protein blot analysis (cropped image). Lane 1—B7H3 Recombinant protein, 2—RB tumor protein lysate, 3—Weri-RB1 protein lysate and 4—Mio-M1 protein lysate; (i) Probed with anti-B7H3 Rabbit monoclonal antibody, (ii) Probed with biotinylated VRF-HS_B7H3-03 aptamer and (iii) & (iv) Probed with GAPDH mouse monoclonal antibody. Results of an aptamer blot from a nonreducing SDS polyacrylamide gel in which B7H3 was clearly detected near 90 kDa similar to antibody. However, the recombinant protein manufacturer of the B7H3 (R&D systems) reported a molecular weight of 38–48 kDa for its product which is consistent with the strongly detected band’s weight (Full raw blot included in Supplementary data Fig. S5).

The binding of five B7H3 aptamers (FITC and biotin labelled) to recombinant B7H3 protein, protein lysates of RB tumor, Weri-RB1 cell lysate, and Weri-RB1 cell secretome was analysed by Dot-blot. All five aptamers were able to bind B7H3 in the samples analysed but didn’t show binding to BSA or secondary control (Fig. 4a). Due to the variable amount of B7H3 present among the samples, it showed variation in the intensity of staining both in FITC and biotinylated aptamers.

#### Sandwich dot bot

The Sandwich dot blot was performed to determine if the aptamers bind to different epitopes of the B7H3 protein, wherein one of the aptamers can be used as a capture and another aptamer for the detection so that an assay similar to sandwich ELISA can be developed. The unlabelled aptamers were used as capture aptamers which were used to bind and capture the B7H3 protein in various samples analysed. After binding of the B7H3 protein by the capture aptamer, the protein was detected by other aptamers which were either tagged by FITC or biotin. We have tried different combinations with the five aptamers wherein the aptamers VRF-HS_B7H3-01 and VRF-HS_B7H3-03 were found to be better combinations in either way and can be coupled for the sandwich assay (Fig. 4b). This aptamer pair could bind different epitopes of the recombinant B7H3, B7H3 of the RB tumor, Weri-RB1 cell lysate, and even in the secretome but didn’t show binding to BSA.

#### Western Blotting

We have checked the ability of the five B7H3 aptamers to detect the denatured B7H3 protein by Western blotting using biotin-labelled aptamers. Out of 5 aptamers only one aptamer (VRF-HS_B7H3-03) was able to detect the denatured B7H3 of recombinant protein, RB tumor, and Weri-RB1, whereas it didn’t show the expression in the Mio-M1 cell lysate (Fig. 4c, full blot Fig. S5). We have obtained similar results with that of the B7H3 antibody. Although other aptamers have demonstrated high-affinity and high-specificity binding toward B7H3 proteins on cell surface and in solution, their performances in Western blotting were far from satisfactory.

#### Immunohistochemistry

**Figure 5:**
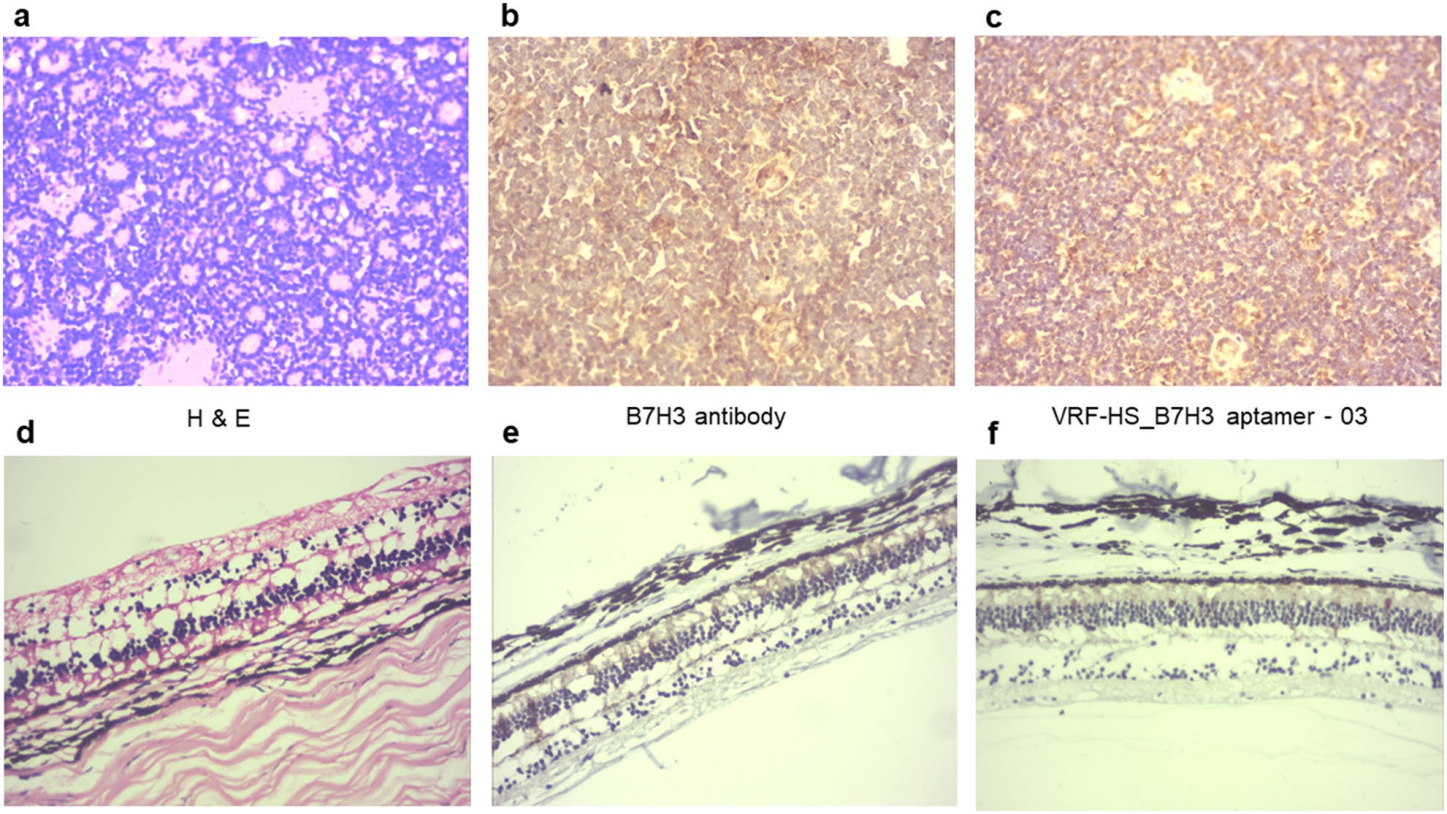
Immunohistochemistry of B7H3 antibody and VRF-HS_B7H3-03 to RB tumour sections and retina. (**a**,**d**) H and E staining, (**b**,**e**) IHC with B7H3 antibody, and (**c**,**f**) IHC with biotin-labelled VRF-HS_B7H3-03 of primary retinoblastoma tumour and retina respectively.

The H&E staining was performed on the RB tumor and retina sections for cellular morphology (Fig. 5a,d). The immunohistochemistry with B7H3 antibody and biotin-labelled VRF-HS_B7H3-03 aptamer on RB tumour showed significant binding to tumour cells which showed a similar pattern as that of B7H3 antibody (Fig. 5b,c) with no background or non-specific binding. The aptamer or antibody showed negligible binding to the retina (Fig. 5e,f).

## Discussion

In this work, our goal was to identify the aptamer that could be applied for multiple assays as an alternative to antibodies. The antibody-based platform is employed to detect numerous targets, but it is consistently hindered by factors such as the time required for production, stability, the impact of modifications, and reproducibility. Even when rigorously validated antibodies are available, the use of multiple antibodies specific for each application and application-specific validation to assess the functionality and target accessibility adds to the complexity and cost of experimental protocols^27^. Further, for any longitudinal studies or clinical diagnostic development, the uninterrupted supply of affinity reagents is very much considered for maintaining reproducibility for consistent sensitivity and specificity of targets intended^28^. When supply is interrupted or halted without warning^29^, the speed of research progress is impeded. The pursuit of alternatives to antibodies is an actively researched field and holds immense importance. Aptamers are gaining increasing attention in antibody-based applications due to several potential performance advantages they offer.

We employed a hybrid-SELEX protocol for DNA aptamer production that recognizes B7H3, a member of the B7 family proteins. Numerous variations in aptamer production protocols have been described, with hybrid-SELEX combining the advantages of both cell SELEX and purified protein-based SELEX^30^. The success of hybrid-SELEX primarily relies on selecting an appropriate starting library. Our innovative approach involved using CSEP-15 as the initial library. Building upon our prior research on B7H3 expression in retinoblastoma and B7H3 antigen density on Weri-RB1 and Mio-M1 cell lines, we screened the retinoblastoma cell-SELEX enriched pools to assess their binding affinity with recombinant B7H3 protein. Notably, CSEP-15 exhibited the highest fluorescence intensity in this context. Furthermore, we have taken advantage of the benefits of magnetic beads for immobilizing His6-tagged protein targets during selection. This strategy promotes a uniform and proper orientation of proteins on the bead surface, and ease in removing the unbound sequences. For the selection of highly efficient aptamers, each cycle of protein-SELEX was followed by a cycle of cell-SELEX. The flow cytometry and dot-blot analysis of the odd rounds of the ssDNA pool showed that the fluorescence intensity increased significantly in the seventh round, indicating the successful enrichment of an aptamer species. Overall, sequences with high binding affinities for the B7H3 were gradually enriched, and no significant improvement was observed when the number of selection rounds was greater than seven. Therefore, the enriched ssDNA pools were selected for sequencing. After sequencing, the top ten sequences with the highest number of reads in the sequencing pools with variable sequences were identified and phylogenetic analysis revealed that sequences were distributed into five groups. The five candidate aptamers (VRF-HS_B7H3-01 to VRF-HS_B7H3-05) one from each group were selected for further characterization.

We have demonstrated the functional versatility of the top five B7H3 aptamers through their application in various assays, comparing their performance with that of the B7H3 antibody. The relative binding affinities of the aptamers were first assessed by Flow cytometry. This sort of initial screening can help prioritize specific aptamers for further comprehensive characterization and determination of apparent dissociation constant. We have used PE-conjugated antibodies for comparison to FITC labelled aptamers, as the only fluorophore that currently conjugates to an antibody in a 1:1 ratio is phycoerythrin^31^ whereas aptamers can be conjugated with any fluorophore with a 1:1 stoichiometric ratio of aptamers to the fluorophore. All five FITC labelled aptamers showed a similar binding affinity with that of the PE-conjugated antibody to Weri-Rb1 cells showed no binding to Mio-M1 cells (Fig. S3) and had a Kd value in the nanomolar range. The aptamers with lower Kd values showed a similar binding percentage as that of an antibody to both Weri-Rb1 and Mio-M1. We have obtained 70–80% of binding by flow-cytometry of aptamers after 24 h at room temperature in ABB similar to 65% of aptamer cell-binding capacity in a comparative assay of antibody vs aptamers against CD4 molecules^32^. In addition to their higher stability to degradation, aptamers are smaller in size approximately 15 kDa–25 kDa when compared to 150 kDa of antibodies making them preferable probes for intra-cellular staining in flow-cytometric applications. Besides these advantages, aptamers can be conjugated with nano-particles^33,34^ or make them multivalent^35,36^ thereby enhancing the sensitivity and the performance of the aptamers in flow-cytometric applications. Apart from the above-mentioned advantages the aptamers can be synthesized at low cost when compared to antibodies. The cost per assay with the fluorophore-labelled CD4 aptamer, which was used as a flow cytometric probe in multi-coloured cell-phenotyping, was about 0.002 dollars per assay, whereas the cost per assay of CD4 antibody was 2 dollars^37^.

The primary utility of a high-affinity probe lies in its ability to identify a target protein within a protein complex. In this context, we conducted dot-blot and sandwich dot-blot or hybrid apta-blotting sandwich assays as an alternative to ELISA. These were done to investigate the potential of aptamers to function as specific and sensitive probes for detecting the target protein in both protein complexes and secretory forms. All the biotinylated B7H3 aptamers developed demonstrated qualitative binding to B7H3 in tumor and cell lysates, including the secretory form of the B7H3 protein. These bindings exhibited variable concentrations across different samples compared to the recombinant protein and showed similar results to that of the antibody. We extended our investigation of aptamer-based sandwich assays to further assess B7H3 aptamers using both "Capture aptamer" and "Reporter aptamer" and successfully achieved B7H3 recognition by utilizing a matched aptamer pair, namely VRF-HS_B7H3-01 and VRF-HS_B7H3-03 with non-overlapping binding sites. We meticulously analyzed binding specificity, cross-reactivity between the capture and reporter aptamers, as well as background signal noise. This analysis involved both specific proteins (recombinant B7H3 protein, tumor lysate, cell lysate, secretome) and nonspecific proteins (BSA). A key advantage of the sandwich assay is enabling multiple target-based outputs, adaptability to expanded formats with analytical modifications like ELONA (ELISA with aptamers), and developing a point-of-care lateral flow In-vitro diagnostic (IVD) kit.

We conducted a western blot analysis to investigate the feasibility of using the developed aptamers for detecting the denatured form of B7H3. Among the five screened aptamers, only one successfully detected B7H3 in the Western blot. This outcome highlights that the denaturation or immobilization of the target can lead to the loss of potential binding sites for aptamers. Compared with conventional antibody-based immunoblotting, such aptamer-based procedure was able to selectively label target protein in a complex mixture and was more time-effective and cost-effective.

Aptamers have emerged as a novel category of molecular probes, finding applications in diagnostic pathology as substitutes for antibodies in immunohistochemistry. They facilitate precise visualization of pathological conditions^11,38^. The smaller size of aptamers offers advantages such as improved staining in dense tissues and the ability to stain co-localized proteins, unlike antibodies, which might face steric hindrance due to their larger size. Apta-histochemical staining using B7H3 aptamers predominantly targeted tumor cells and specificity was confirmed by comparison with antibody-based staining. The results indicated that the aptamer exhibited a staining pattern similar to that of the B7H3 antibody, while offering simpler reaction conditions, including reduced incubation time and fewer washing steps. The major limitations of the present study are:

(i) Aptamer has been compared to a single commercial antibody highly reported in the literature. In the future, it will be beneficial to compare it with a range of B7H3 antibodies generated through different methods and raised in different species and formats to widen the scope of applications.

(ii) The specificity of the aptamer has not been extensively validated using other B7 family proteins. However, western analysis of the RB tumor lysate known to express other B7 family proteins showed specificity to B7H3.

## Conclusion

In this study, DNA aptamers were generated by an *in-vitro* selection that selectively recognized B7H3 protein in native, denatured, and secretory forms. Direct comparison of aptamer with conventional antibody-based assays demonstrated that these aptamers (VRF-HS_B7H3-01 to VRF-HS_B7H3-05) can be applied in flow-cytometry, IHC, dot-blot, hybrid apta-blotting sandwich assays, and western blotting. This work suggested that the methodology developed here by the generated aptamers can be an effective and low-cost alternative to conventional B7H3 antibody-based assays. Further studies in standardizing these assays, especially in dynamic and varied experimental conditions, and across a broad dynamic range are crucial for establishing aptamers as reliable alternatives in protein detection and quantification.

## Materials and methods

### Ethics statement

The study was conducted in compliance with the declaration of Helsinki approved by the Institutional Ethics committee of the Vision Research Foundation, (Ethics Number: 563-2016-P) Chennai, Tamil Nadu, India.

### Cell lines and buffers

Retinoblastoma cell-line, Weri-RB1 was purchased from Riken and a non-cancerous Muller glial cell-line, Mio-M1 was derived from the neural retina and was a gift from Dr. Professor Astrid Limb (UCL, Institute of Ophthalmology, London, England). Weri-RB1 cells were cultured in RPMI medium and Mio-M1 in DMEM. All the cell-culture media are supplemented with 10% FBS, 100U penicillin/ml, 100 μg streptomycin/ml and 0.25 μg amphotericin B/ml and cells were cultured in a humidified 5% CO_2_ incubator at 37 °C. Cell-culture media and supplements are from Gibco (Thermo-Fisher Scientific, IN). All experiments with cells were done with 80% confluent cultures within 2–4 passages.

### Buffers

Aptamer wash buffer (AWB): 25 mM Glucose (Sigma), 0.9 mM CaCl_2_, and 5 mM MgCl_2_ in Dulbecco’s PBS without calcium and magnesium (Gibco).

Aptamer binding buffer (ABB): AWB with 0.1 mg/ml yeast tRNA (Invitrogen), 0.1 mg/ml Salmon sperm DNA (Ambion, Life technologies), 5% FBS (Gibco) and 1 mg/ml Bovine Serum Albumin (BSA) (Sigma).

Bead binding buffer (BBB): 100 mM Sodium Phosphate (pH 8.0), 600 mM NaCl, 0.02% Tween-20.

Bead suspension buffer (BSB): 6.5 mM Sodium phosphate (pH 7.4), 140 mM NaCl, 0.02% Tween-20.

#### Protein and target beads preparation

To select high-affinity aptamers against B7H3, the recombinant human B7-H3 His-tag (R&D 1949-B3-050) protein, and Dyna-beads His-tag isolation and pulldown beads (Life technologies 10103D) were used. Target beads were prepared by washing 5 mg of Dyna-beads in BBB and incubated with 50 µg of his-tagged B7H3 recombinant protein following the manufacturer’s instructions and suspended in BSB and stored at 4 °C.

### ssDNA library selection and primers

The initial library was selected by screening the binding affinities of enriched pools of RB cell-SELEX (Maradani et al.^14^) on recombinant B7H3 protein by dot-blot. Briefly 10 ng of the recombinant B7H3 was blotted on to the methanol activated PVDF membrane and allowed to bind. The protein blots were blocked with ABB for 1 h and washed thrice with AWB. After washing the blots were incubated with FITC labelled library pool, 5th, 10th, 15th, 20th, 22nd, 24th, and 26th enriched RB cell-SELEX pools dissolved in ABB for one hour, washed thrice with AWB and imaged with image scanner. The 15th pool of RB cell-SELEX named as Cell-SELEX enriched pool–15, CSEP-15 was selected as initial library for B7H3 hybrid SELEX. The library contains 40 bases (5ʹ-AGGGAAGAGAAGGACATATGAT—40N—TTGACTAGTACATGACCACTTGA-3ʹ) randomized sequences. The FITC-labelled forward primer (5ʹ-TAGGGAAGAGAAGGACATATGAT-3ʹ) and a biotinylated reverse primer (5ʹ-TCAAGTGGTCATGTACTAGTCAA-3ʹ) were used in PCR for the synthesis of dsDNA. The primers and aptamers were synthesized by Integrated DNA Technologies (IDT).

#### Hybrid-SELEX process

The hybrid-SELEX procedure was performed as described by Hassan et al. 2017^39^ with minor modifications. Each round of hybrid SELEX includes one cycle of protein SELEX followed by one cycle of cell-SELEX.

For the first cycle of protein-SELEX, 100 pmol of CSEP-15 was denatured by heating at 95 °C for 5 min, snap-cooled on ice for 3 min, and incubated with pre-washed 3 × 10^5^ empty dyna-beads for 30 min. The negative selection with empty dyna-beads was performed to remove the ssDNA strands which may bind to dyna-beads. The unbound ssDNA was collected and incubated with 5 × 10^5^ immobilised His-tagged B7H3 target beads for 2 h on a rotary shaker for the positive selection. After incubation beads were washed to remove unbound or weakly bound sequences and added to the PCR master-mix to be amplified. PCR was carried out using FITC- and biotin-labelled primers. The strand separation of the PCR product was performed by streptavidin-coated sepharose beads (GE Healthcare, USA) followed by desalting with a NAP-5 column (GE Healthcare); the generated FITC-labelled ssDNA pool was lyophilized and used as a pool for following the cell-SELEX cycle.

For the cell-SELEX cycle, the pool generated from protein-SELEX was denatured and first incubated with 1 × 10^7^ Weri-RB1 cells on ice for two hours with gentle agitation. After incubation, cells were collected and centrifuged at 1000 rpm for 3 min at 4 °C. The sequences bound to Weri-RB1 cells were recovered by heating at 95 °C for 15 min, cooled on ice for 5 min, and eluted into 500 ul of deionized water by centrifugation at 14,000 rpm for 5 min. The ssDNA pool was generated from the recovered sequences by PCR amplification followed by strand separation as above and incubated with 1 × 10^6^ Mio-M1 cells at 4 °C for 30 min with gentle agitation. The unbound ssDNA sequences were then separated by centrifugation at 14,000 rpm for 5 min and used as a pool for the next cycle of protein-SELEX.

To acquire sequences with high affinity and specificity, we enhanced the strength and the frequency of washes (Table 2). Additionally, the counter selections (empty beads and Mio-M1 cells) were also gradually increased to reduce the nonspecific binding. A total of 9 rounds of selection were performed before sending the pools for sequencing.

**Table 2:** Increasing the stringency of selection conditions in hybrid-SELEX process.

Cycle	Protein—SELEX		Cell—SELEX		Time of incubation (positive selection)	Time of incubation (negative selection)	Number of washes
	Target beads	Empty beads	Weri-RB1 cell number	Mio—M1 cell number			
1	5 × 10^5^	3 × 10^5^	1 × 10^7^	1 × 10^6^	120	30	1 × 3 min
3	4.5 × 10^5^	3.5 × 10^5^	0.75 × 10^7^	0.25 × 10^7^	120	60	2 × 3 min
5	4 × 10^5^	4 × 10^5^	0.5 × 10^7^	0.5 × 10^7^	60	60	3 × 2 min
7	3.5 × 10^5^	4.5 × 10^5^	0.25 × 10^7^	0.75 × 10^7^	60	120	3 × 3 min
9	3 × 10^5^	5 × 10^5^	1 × 10^6^	1 × 10^7^	30	120	3 × 3 min

The enrichment progress was monitored by incubating 250 nM of FITC-labelled ssDNA pools with the 2 × 10^5^ Weri-RB1 and Mio-M1 cells (dissociated with non-enzymatic dissociation buffer) in 500 μl of ABB at 4 °C for 30 min after washing once with 1X PBS and twice with AWB. After incubating with the enriched pools, the cells were washed three times with WB and analysed by flow cytometry (BD FACS Lyric). The FITC-labelled initial ssDNA library was used as a negative control. All experiments were repeated three times. After confirming the saturation of enrichment by FACS, the enriched pools were sent for sequencing to Base Pair Biotechnologies (Texas, US). The top five aptamer sequences from the NGS results are further evaluated.

#### Binding assays by flow cytometry

The binding assays of the B7H3 aptamers were performed by flow cytometry. To evaluate the binding efficiency, 5 × 10^5^ Weri-RB1 and Mio-M1 cells were washed with PBS and blocked with ABB on ice for 30 min. After blocking the cells are incubated with 250 nM of FITC-labelled aptamers dissolved in ABB for one hour on ice in the dark. Cells were washed three times with 0.5 ml of AWB, resuspended in 0.5 ml of 1X PBS or ABB, and analysed by the BD FACS Lyric cytometer. The initial FITC-labelled ssDNA library and unstained cells served as controls.

The equilibrium dissociation constant was determined, both by using cells and recombinant protein. Briefly, varying concentrations (0 to 500 nM) of FITC-labelled aptamers in 200 μl of ABB were added to 5 × 10^5^ Weri-RB1 and Mio-M1 cells and to the membranes where 10 ng of recombinant B7H3 protein was spotted and incubated for 60 min with gentle shaking in the dark. Cells were then washed thrice with 0.5 ml of AWB, re-suspended in 0.5 ml of ABB, and analysed by flow cytometry. The membranes were washed with 0.5 ml of AWB and imaged by Chemi-doc. The ssDNA library was used as the negative control. The mean fluorescence intensity of each sample was subtracted from the mean fluorescence intensity of the background produced by the ssDNA library. The equilibrium dissociation constant (Kd) of the aptamer–cell interaction was obtained using GraphPad Prism (GraphPad Prism version 7.00 for Windows) by fitting the dependence of fluorescence intensity of specific binding on the concentration of the aptamers to the equation:

$$Y=Bmax\times X/(Kd+X)$$

where Y is fluorescence intensity of cells, X is concentration of the aptamer and B_max_ is maximum binding potential^14^.

#### Dot blot

Whole-cell lysates were prepared from the RB tumor, Weri-RB1, and Mio-M1 cells with RIPA (Sigma-Aldrich R0278) containing Protease Inhibitor Cocktail (Sigma-Aldrich P2714). The proteins were quantified by a BCA protein assay kit (Thermo Scientific, A53225). 10 ng of B7H3 recombinant protein, 10 µg of RB tumor protein lysate, 10 µg of Weri-RB1 cell line protein lysate, 5% of BSA, 50 µl of Weri-RB1 cell secretome and binding buffer as secondary control were spotted on the methanol activated PVDF membrane (GE Healthcare, Little Chalfont, UK) and allowed for proteins to bind to membrane. After blotting the membrane was blocked with ABB for aptamers for one hour. After blocking the membrane was incubated in 100 nM of FITC or biotinylated labelled B7H3 aptamers for one hour. After incubation the membrane was washed three times with TBST and directly imaged in the case of FITC-labelled aptamers. In the case of biotinylated aptamers, the membrane was incubated with streptavidin HRP (Molecular probes S-911, 1:10,000 dilution) for 30 min followed by chemiluminescent (Biorad; catalogue No: 1705060) substrate and imaged by Chemi-doc.

#### Sandwich dot blot

The sandwich dot blot was performed on a nitrocellulose membrane (GE Healthcare, Little Chalfont, UK). In the first step, the membrane was incubated with 500 nM of one of the unlabelled B7H3 aptamers for one hour. After incubation, the membrane was washed thrice with AWB and blocked with ABB for 30 min. After blocking, 10 ng of B7H3 recombinant protein, 10 µg of RB tumor protein lysate, 10 µg Weri-RB1 protein lysate, 50 µl of Weri-RB1 cell secretome, and 5% BSA were spotted onto the membrane and allowed to bind. After proteins were bound, the membrane was blocked again with ABB for 30 min and incubated for 60 min with 250 nM of FITC or biotin labelled B7H3 aptamers other than the aptamer used for blocking the membrane and processed the same as dot-blot.

#### Western blotting

10 ng of recombinant B7H3 protein and 20 µg of proteins of Rb tumour, Weri-RB1, and Mio-M1 cell lysates were denatured in lamelli buffer by heating for 10 min at 95 °C. Denatured proteins were loaded and separated by 12% SDS-PAGE and transferred to the methanol-activated PVDF membrane.

In aptamer-based protein blotting, the membrane was first blocked with ABB (without FBS) for 30 min at room temperature. The membrane was then incubated with 250 nM of biotinylated B7H3 aptamers for 60 min at 4 °C and washed thrice with AWB. After washing, the membrane was incubated with streptavidin HRP (Molecular probes S-911, 1:10,000 dilution) for 30 min and washed thrice with AWB. The bands in the membrane were detected with clarity western electrochemiluminescence (ECL) substrate (Biorad; catalog No: 1705060) under Biorad MP ChemiDoc Imaging systems.

In the conventional antibody-based Western blot, the PVDF membrane was first blocked with 5% dried milk for one hour at room temperature. Membranes were incubated in primary antibodies against B7H3 rabbit monoclonal antibody (Cell Signaling technology, 14,058, 1:1000 dilution), in TBST containing 1% BSA overnight at 4 °C. Membranes were washed with TBST and incubated with anti-rabbit IgG HRP conjugated secondary Antibody (Cell Signaling technology,7074S, 1:1000 dilution) 1% BSA for 1 h at room temperature and the bands were detected by ECL substrate as above.

For both the protocols GAPDH mouse monoclonal antibody (Cell Signaling technology, 97166, 1:1000 dilution) and anti-mouse IgG HRP conjugated secondary antibody (Cell Signaling technology, 7076, 1:1000 dilution) in TBST containing 1% BSA was used as a loading control.

#### Immunohistochemistry

Immunohistochemistry was performed to assess the binding of B7H3 aptamer (VRF-HS_B7H3-03) to RB primary tumor as a positive control, lysed blood spiked with Weri-RB1 cells (leukocytes as a negative control), and cross-validated with IHC using B7H3 antibody (Cell Signaling technology, 14058, 1: 100 dilution). The FFPE sections were deparaffinized and antigen retrieval was done by a standard procedure using 0.01 M citrate buffer (pH 6.0). IHC with aptamers was carried out as mentioned in Maradani et al.^14^. For IHC with the antibody, antigen-retrieved sections were blocked with HRP blocker (Dako K8023) and washed three times with PBS. Slides are probed with B7H3 antibody overnight and washed with PBS thrice. After washing the slides are incubated with HRP plus (Dako K8023) for 30 min at room temperature. After washing, the slides were stained with DAB for 5 min, counterstained with hematoxylin for 1 min, and mounted and analysed under the light microscope (Nikon Eclipse Ci-L, Tokyo, Japan).

### Supplementary Information

Supplementary Information.

## Data Availability

Data and aptamer sequences were uploaded at NCBI and can be downloaded using the accession number PRJNA844177.
