# Supplementary material for: Development of DNA aptamers targeting B7H3 by hybrid-SELEX: an alternative to antibodies for immuno-assays
Source: Sci Rep. 2024 Jun 12;14:13552. doi: 10.1038/s41598-024-64559-7 (PMC11169341; doi:10.1038/s41598-024-64559-7)

Development of DNA aptamers targeting B7H3 by hybrid-SELEX – an alternative to antibodies for immuno-assays

Bhavani Shankar Maradani[1,3], Sowmya Parameswaran[2], Krishnakumar Subramanian[1*]

*Corresponding author: drkk@snmail.org, drkrishnakumar_2000@yahoo.com

Running title: Aptamers for immuno-assays.

Figure S1: Frequency of the top 10 aptamers in the sequenced pools. Apt1 to Apt10 – VRF-HS_B7H3-01 to VRF-HS_B7H3-010 respectively; 5 positive – 5th positive hybrid SELEX round; 7 positive – 7th positive hybrid SELEX round; 9 positive – 9th positive hybrid SELEX round.

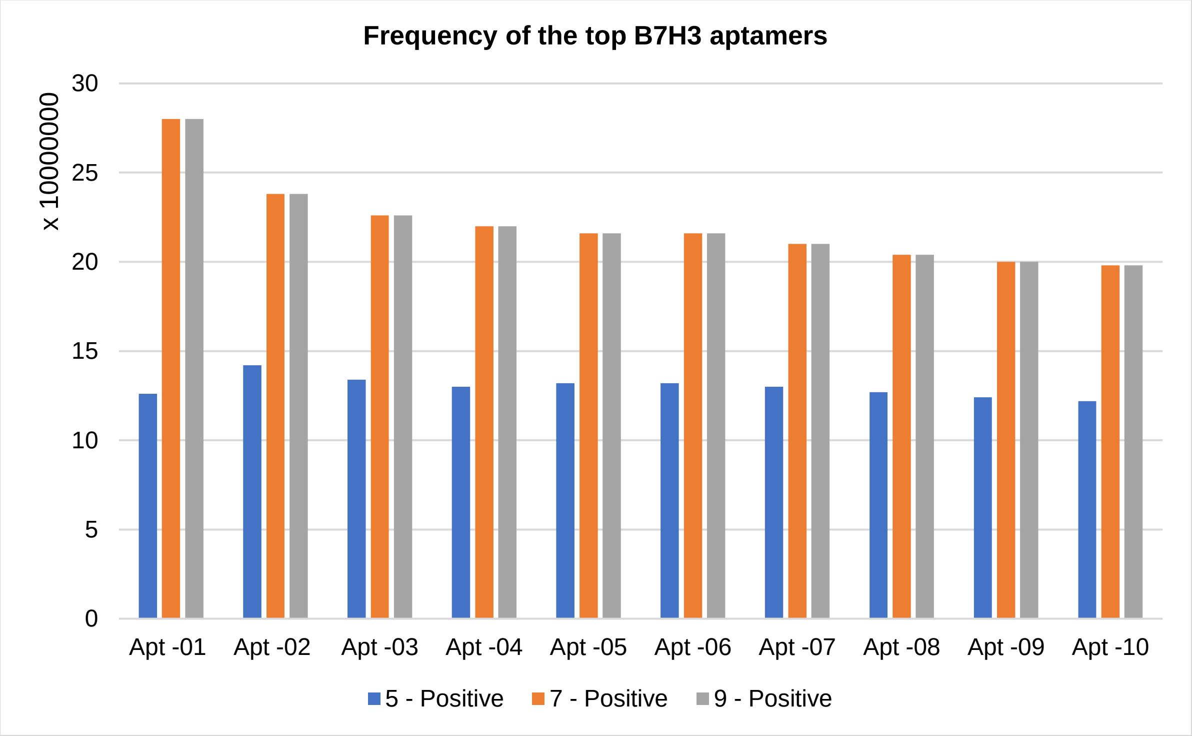

Figure S2: Selection of candidate aptamers by AptaSUITE using the NGS data. a – The distribution of the reads per round. b – The enrichment and count of the top five aptamers from the high throughput sequencing of the positive pools.

a

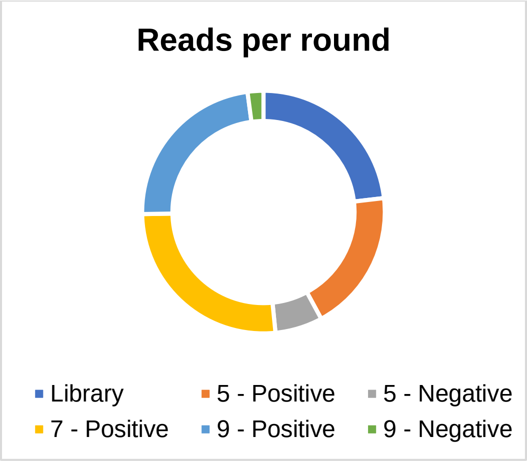

b

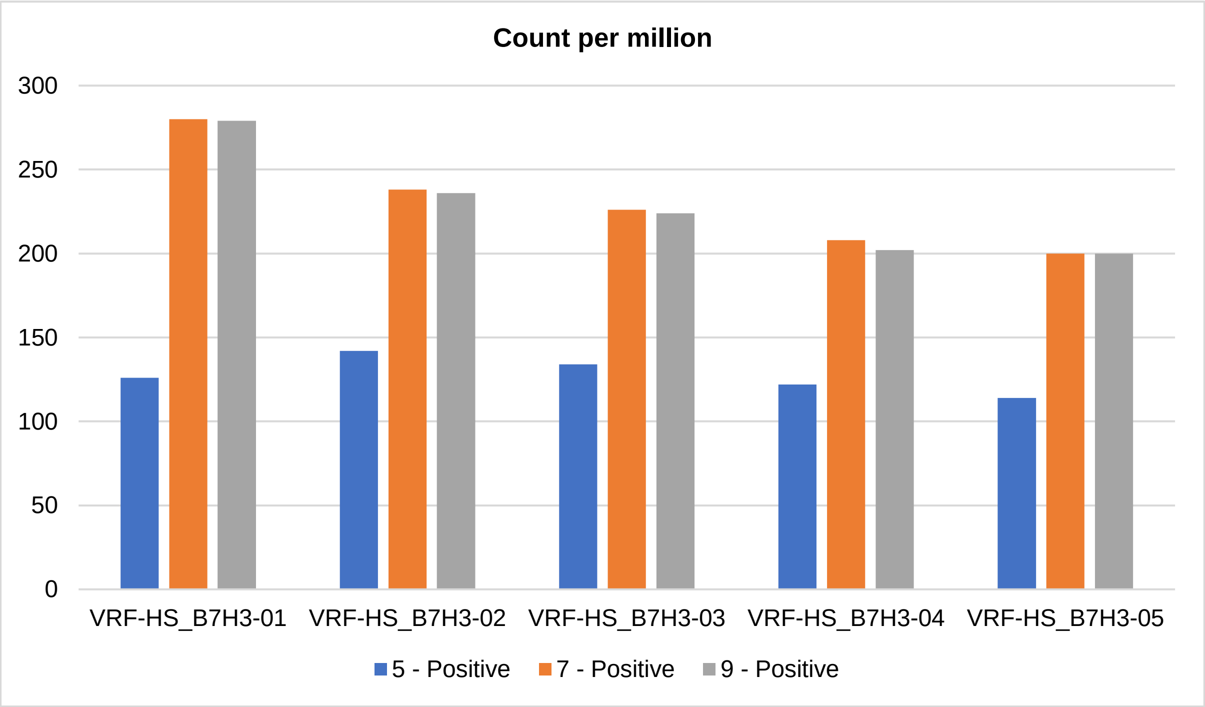

Figure S3: Flow cytometry analysis by PE conjugated B7H3 antibody in Weri-RB1 and Mio-M1 cells and by VRF-HS_B7H3-03 aptamer in PC-3, SCC-25, MCF-7 and A549 cell lines. Flow cytometry analysis with B7H3 antibody revealed B7H3 expression with 97% positivity in (a) Weri-RB1 cell line and absent in (b) MIOM1 cell line. Flow cytometry analysis of with VRF-HS_B7H3-03 aptamer in (c) PC-3, (d) SCC-25, (e) MCF-7 and (f) A549 cell lines.

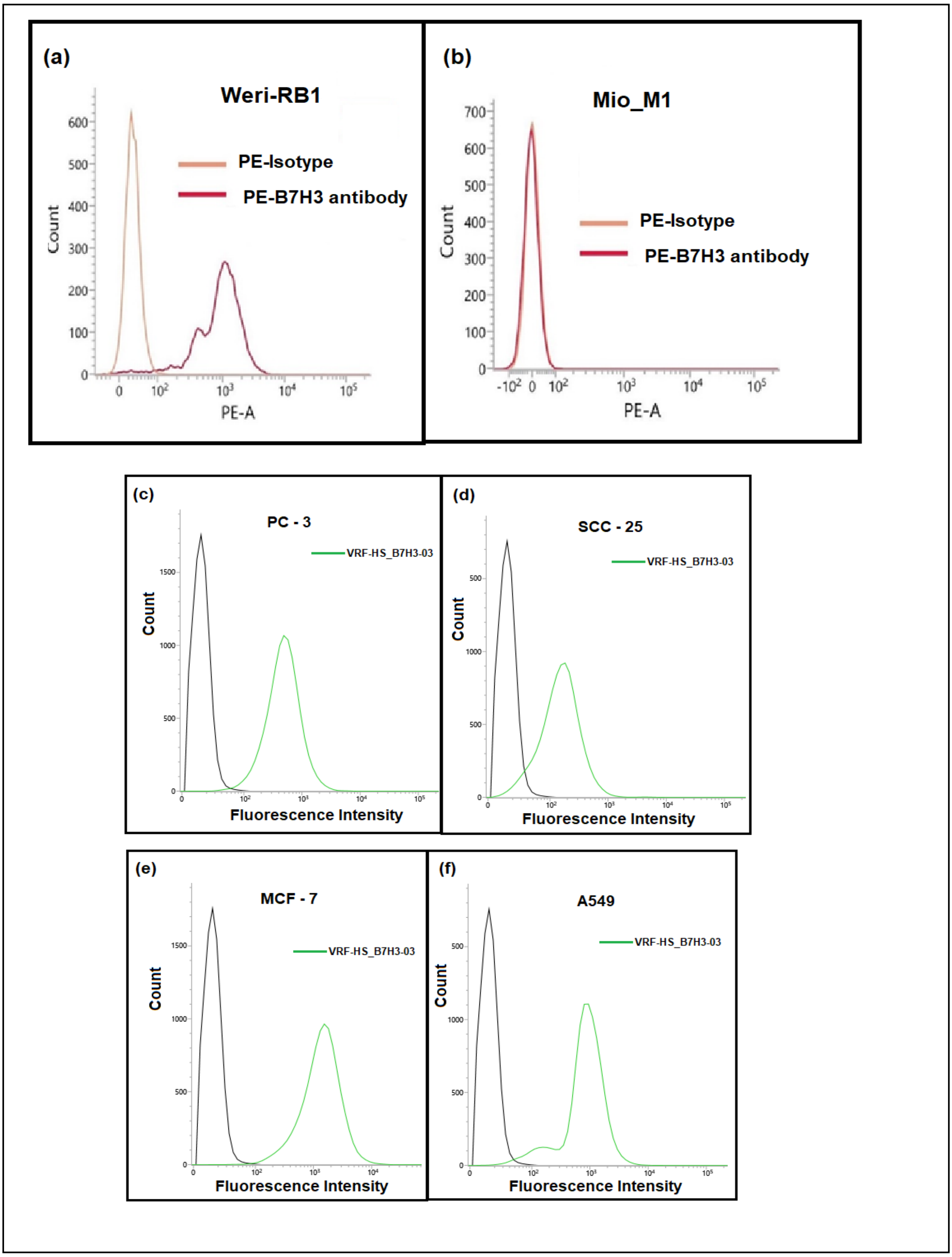

Figure S4: Raw full dot-blot of aptamers with FITC and biotin labelled B7H3 aptamers. (a) Dot-blot of the top five B7H3 aptamers; (b) Sandwich dot-blot with VRF-HS_B7H3-01 and VRF-HS_B7H3-03 aptamers

a

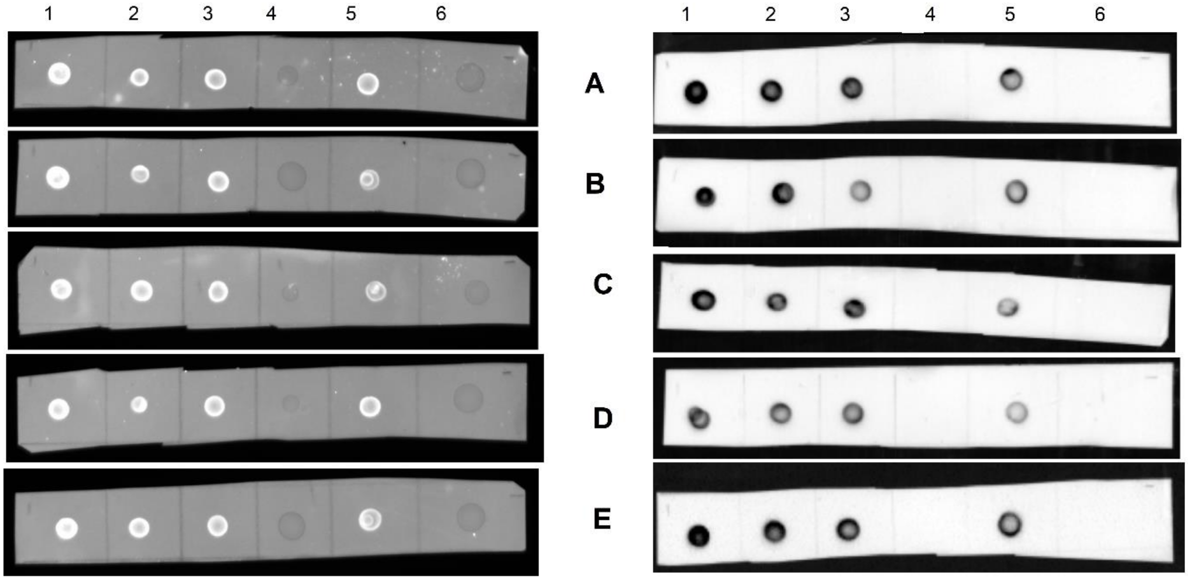

Dot-blot with FITC labelled B7H3 aptamers **Dot-blot with biotinylated B7H3 aptamers**

A - VRF-HS_B7H3-01, B - VRF-HS_B7H3-02, C - VRF-HS_B7H3-03, D - VRF-HS_B7H3-0-4 and E - VRF-HS_B7H3-05

1 - Recombinant B7H3 protein, 2 - RB tumor lysate, 3 - Weri-B1 cell lysate, 4 - BSA (5mg/ml), 5 - Weri-RB1 cell line secretome and 6 - Secondary control

b

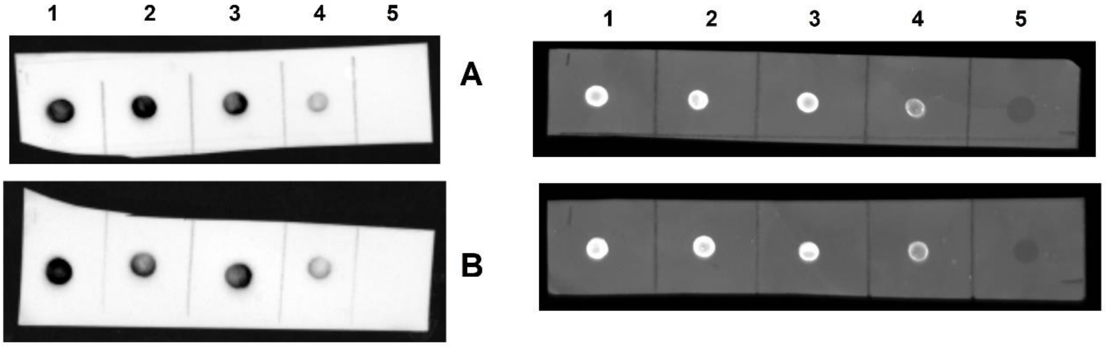

Sandwich dot-blot with biotinylated aptamers **Sandwich dot-blot with FITC labelled aptamers**

A - VRF-HS_B7H3-01 as capture aptamer and VRF-HS_B7H3-03 as detection aptamer.
B - VRF-HS_B7H3-03 as capture aptamer and VRF-HS_B7H3-01 as detection aptamer.

1 - Recombinat B7H3 protein, 2 - RB - tumor lyatse, 3 - Weri-RB1 lysate, 4 - Weri-RB1 cell line secretome and 5 - BSA (5mg/ml)

Figure S5: Raw full western blot of B7H3 antibody, VRF-HS_B7H3-03 aptamer and corresponding GAPDH antibody.

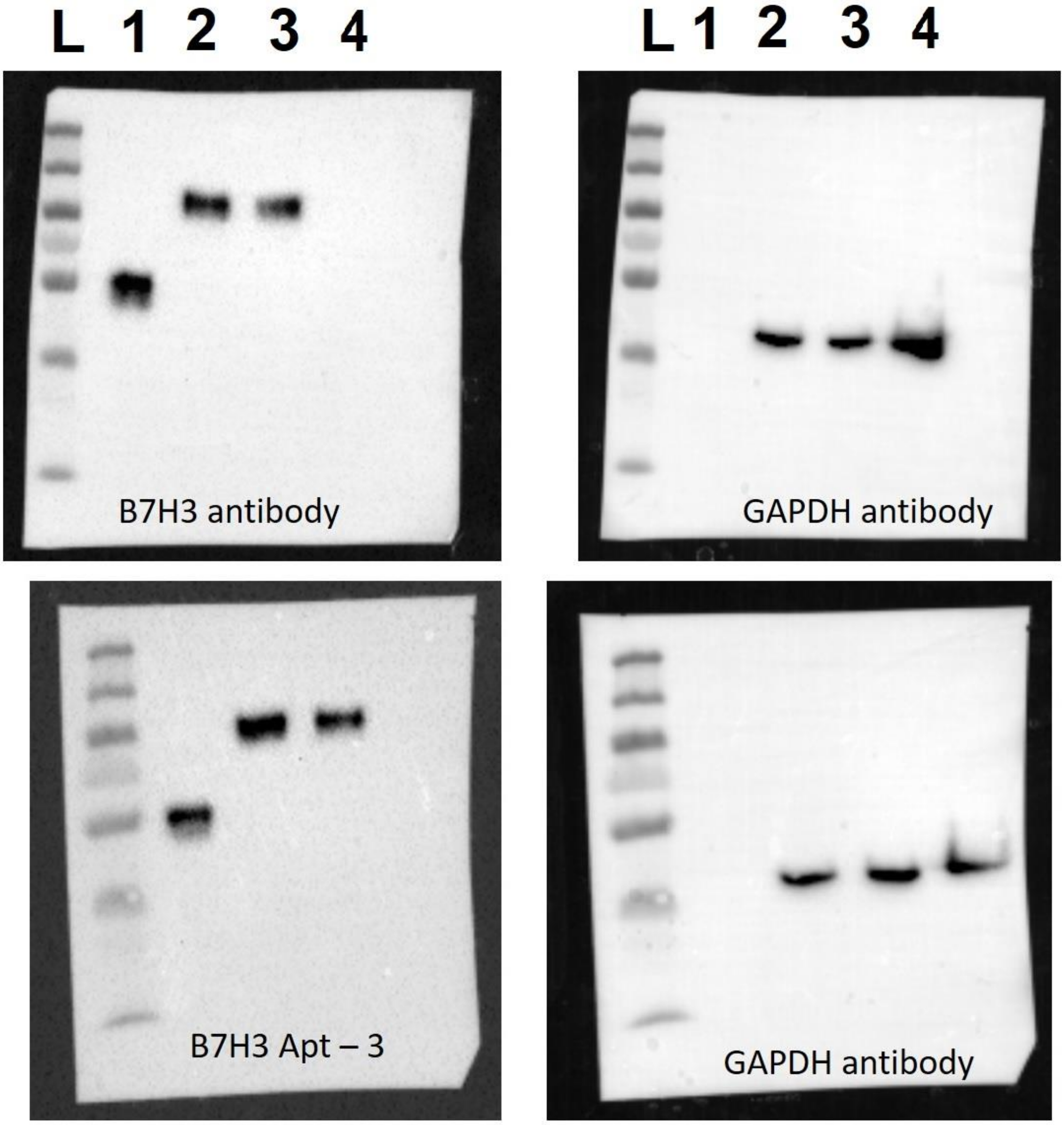

Western blot with B7H3 antibody and VRF-HS_B7H3-03 aptamer

L - Ladder, 1 - Recombinant B7H3 protein, 2 - RB tumor lysate,
3- Weri-RB1 cell lysate and 4 - Mio-M1 cell lysate

Figure S6: The binding affinity of VRF-HS_B7H3-03 aptamer by IHC. (a-c) Breast cancer – positive control and (d-f) Lysed blood sample having leukocytes spiked with Weri-RB1 cells. Black arrow indicated Weri-RB1 cells stained positive with the VRF-HS_B7H3-03 aptamer and leukocytes showing negative. (g-i) sebaceous gland carcinoma of lid, positive for blood vessels (red arrow), and negative for tumor (black arrow). (j-l) Conjunctival squamous cell carcinoma , positive for blood vessels (red arrow), and negative for lymphocytes (blue arrow).

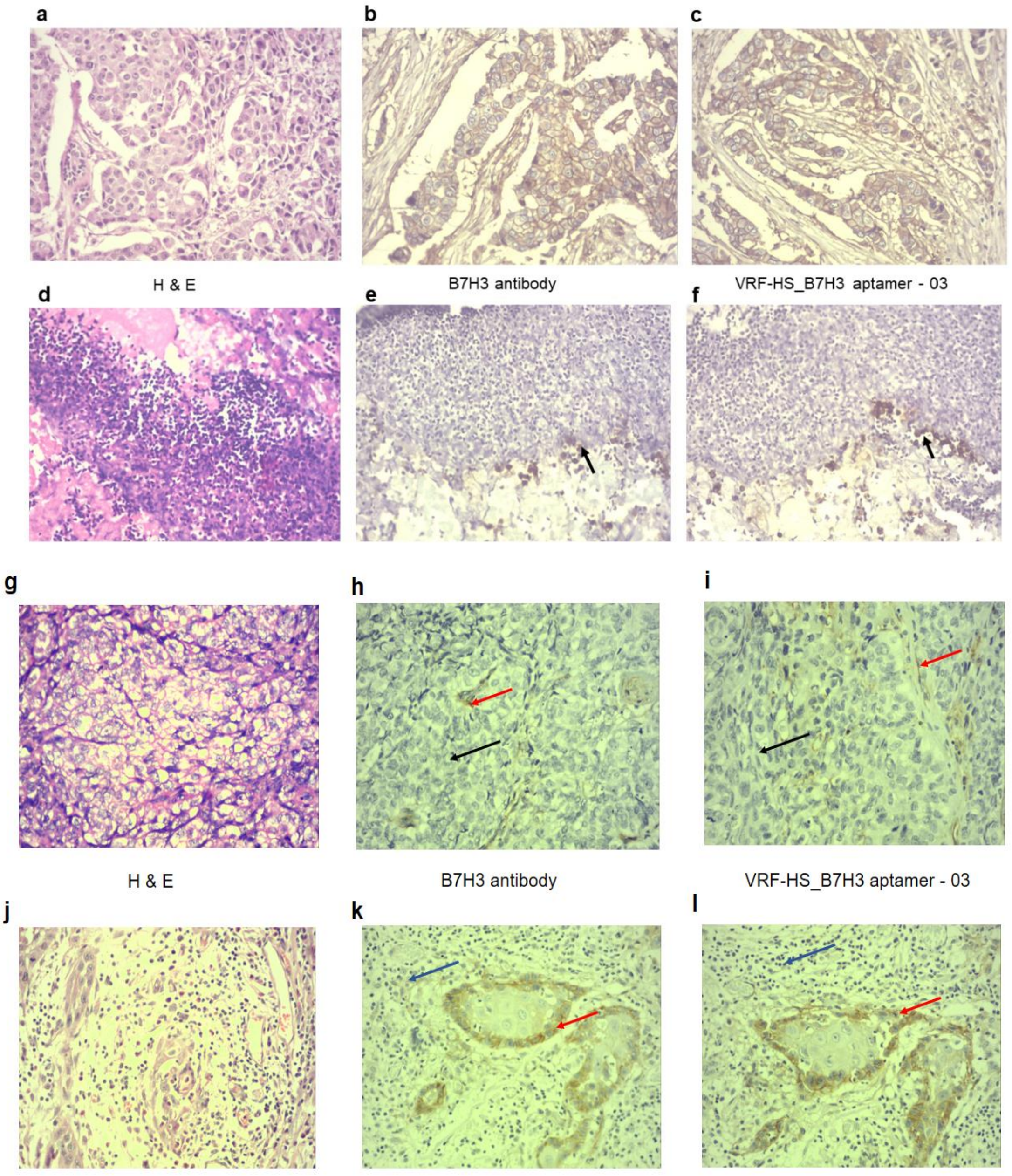

Supplement: Supplementary file 1 — Supplementary Information. [file 41598_2024_64559_MOESM1_ESM.pdf]
